# The New Epidemiology of HIV-Related Kidney Disease

**DOI:** 10.4172/2155-6113.S4-001

**Published:** 2012-01-17

**Authors:** Sandeep K. Mallipattu, Christina M. Wyatt, John C. He

**Affiliations:** 1 Division of Nephrology, Department of Medicine, Mount Sinai School of Medicine, New York, NY, USA; 2 Renal Section, Department of Medicine, James J. Peters VA Medical Center, New York, NY, USA

**Keywords:** HIV, AIDS, HCV

## Abstract

HIV-related kidney disease has been associated with significant morbidity and mortality in the HIV population. It is clear that the epidemiology of HIV-related kidney disease has changed dramatically since the first case reports in 1984. During these early years, the predominant etiology of kidney disease in HIV was recognized as HIV-associated nephropathy (HIVAN), an aggressive form of kidney disease with a high rate of progression to end-stage renal disease (ESRD). Subsequently, with the widespread use of combination antiretroviral therapy (cART), there was a dramatic decrease in the incidence of ESRD attributed to HIV/AIDS. Although the incidence of HIV-related ESRD has plateaued in the last 15 years, the prevalence has continued to increase because of improved survival. Available prevalence estimates do not include HIV-infected individuals with comorbid ESRD, although there is growing evidence that the epidemiology of kidney disease in the HIV-infected population has changed. This article reviews the impact of risk factors such as race, diabetes mellitus, hypertension, hepatitis C virus coinfection, and the chronic use of cART on the changing epidemiology of HIV-related kidney disease. Additionally in this review, we propose potential areas of translational research that will help to further characterize HIV-related kidney disease in the 21^st^ century.

## Introduction

**Figure 1:**
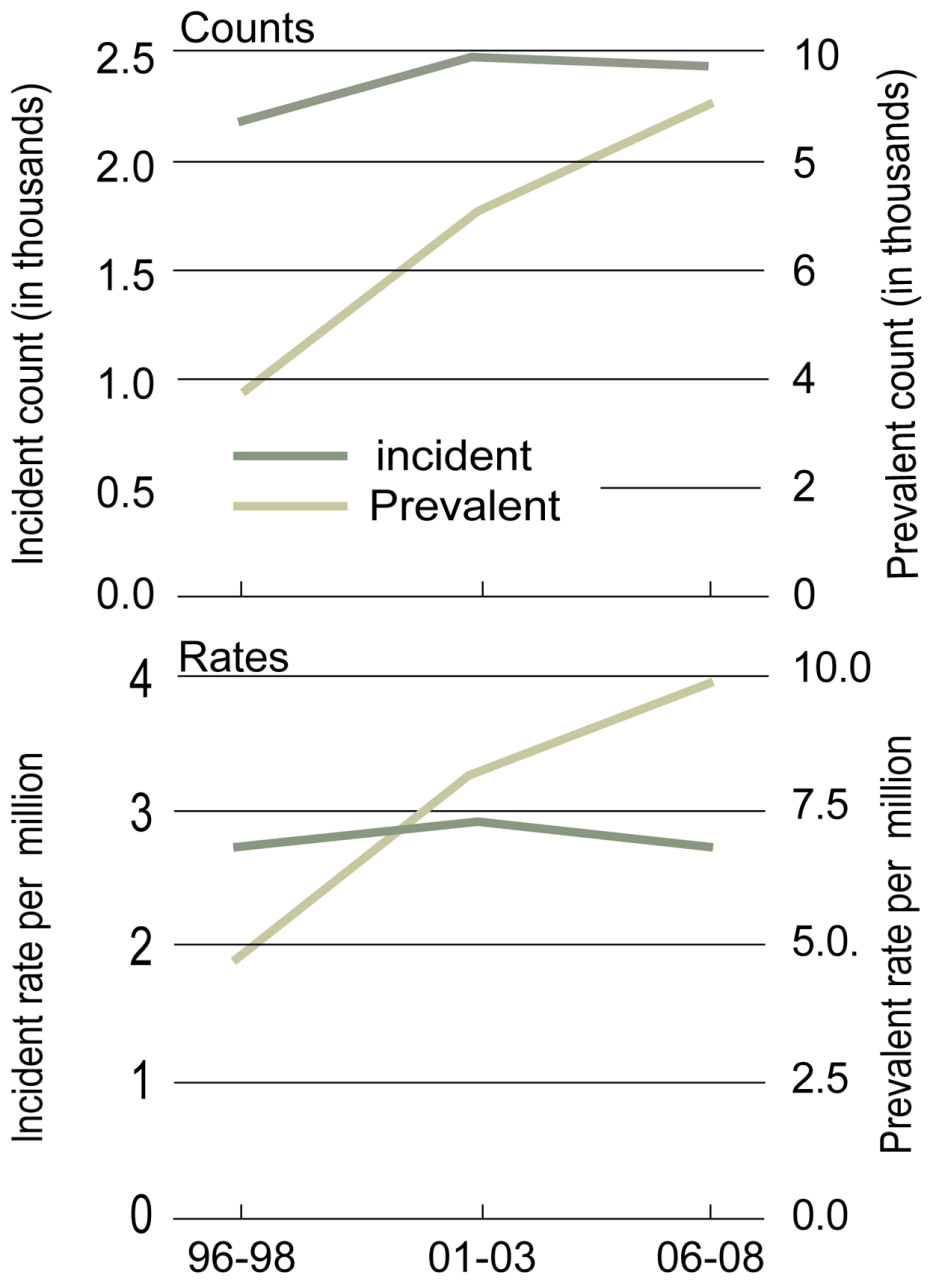
The Incidence and Prevalence of ESRD in patients with AIDS (1996 to 2008). Adapted from 2010 USRDS annual data report

It had been nearly 28 years since the first published case reports of HIV-associated nephropathy (HIVAN), an aggressive form of focal segmental glomerulosclerosis (FSGS) in patients with Acquired Immunodeficiency Syndrome (AIDS) [1–3]. Within years, HIVAN was recognized as not only as an AIDS-defining illness [4], but also a disease predominant in patients of African descent [5]. *In vitro* and *in vivo* models provided clear evidence that local HIV gene expression in the kidney was required for the development of HIVAN [6]. Although HIVAN can result from viral gene expression in podocytes, studies have shown that tubular cells infected with HIV can also play a crucial role in disease pathogenesis [7]. The incidence of HIVAN and the progression to End Stage Renal Disease (ESRD) continued to rise until the advent and the use of combination antiretroviral therapy (cART) in the mid 1990s. Although the widespread use of cART has reduced the incidence of ESRD attributed to HIVAN, the rate of decline has decreased and has in fact plateaued at 800–900 cases per year in the United States [8]. In addition, the prevalence of ESRD in this population has continued to rise due to increased patient survival (Figure 1).

HIV-related kidney disease has lead to a significant burden on health care. For example, in the Women’s Interagency HIV Study, kidney disease was predictive of an increased risk of AIDS defining illness and mortality [9]. Furthermore, decreased kidney function has been associated with increased cardiovascular risk in patients with HIV [10]. Specifically, it was shown that for every 10ml/min/1.73m^2^ decrease in eGFR, there was a 20% increase in the odds of a cardiovascular event [10]. Increased mortality in patients with HIV and chronic kidney disease (CKD) may be partly explained by underexposure and inadequate dose adjustment of cART [11,12]. Finally, the financial and psychosocial repercussions of managing patients on dialysis cannot be neglected.

## The Changing Spectrum of Kidney Disease in HIV

It is clear that the spectrum of HIV related kidney disease has dramatically changed with the widespread use of cART since the mid 1990s. Several studies have shown that many patients that undergo a clinically indicated kidney biopsy are diagnosed with a non-HIVAN related kidney disease in the post-ART era (Table 1) [14–17]. For example, in a cohort of 152 HIV patients with renal biopsies, there was a wide distribution in renal pathology [14]. Although, HIVAN remained the predominant diagnosis on biopsy in this cohort, some of the other key pathological diagnoses included non-collapsing FSGS, acute interstitial nephritis, and diabetic nephropathy in 22%, 8%, and 5% of patients, respectively [14]. In this longitudinal study, the annual proportion of biopsies demonstrating HIVAN significantly decreased from nearly 80% in 1997 to 20% in 2004 [14]. Additionally, a retrospective study from six medical centers in the United States revealed that 53% of HIV patients who underwent a kidney biopsy had a non-HIVAN diagnosis [17]. In individuals without a clinical indication for biopsy, renal pathology from postmortem organ donation in the post-ART era found that arterionephrosclerosis was the most common diagnosis [13]. Studies such as these suggest that the spectrum of kidney disease has considerably changed in the last 15 years. With this change, the clinical course of kidney disease in the cART era has been more indolent, a slow progressive decline in kidney function with lower levels of proteinuria. This indolent course has lead to prolonged time prior to biopsy and has been postulated as a major reason for the delayed diagnosis of non-HIVAN related kidney disease [17]. Finally, in addition to the challenges in diagnosis, the United States Renal Data System (USRDS) no longer collects data on HIV infection as a comorbid condition in incident ESRD patients. This means that nationally representative estimates will be limited to ESRD attributed to HIVAN and will significantly underestimate the burden of ESRD in HIV-infected individuals.

## Definition of CKD in HIV-Related Kidney Disease

CKD has been defined as kidney damage or reduced kidney function that persists for more than three months [18]. Indicators of kidney damage have included an elevation in urinary protein excretion or an estimated glomerular filtration (eGFR) < 60 ml/min. Although eGFR has been widely used to estimate kidney function, none of the currently available estimates have been validated for use in this population. HIV patients are known to have differences in muscle composition that may lead to a reduction in creatinine synthesis, which could impact the performance of creatinine-based GFR estimates. It is also difficult to draw comparisons between studies when different methods have been used to estimate GFR. Studies are ongoing to determine the best method to define CKD in the HIV population.

## HIV and CKD

The estimated prevalence of CKD in HIV has ranged from 2.4–17% across studies (Table 2) [11,19–21,23,29,33,34]. Although there is some variation, most studies have reported an association with older age, black race, hepatitis C virus (HCV) coinfection, type II diabetes mellitus (DM), and exposure to certain ART [20,21].

## HIV with Type II Diabetes Mellitus and Hypertension

In the general population, DM and HTN increase the risk of CKD by 10% and account for 71% of all ESRD cases in the Unites States [18]. In the HIV population, the prevalence of DM and HTN has been reported as 8% and 20%, respectively [22]. The widespread use of certain cART regimens may have contributed to the increased incidence of DM [24–26], which has been specifically attributed to protease inhibitors and non-nucleoside reverse transcriptase inhibitors [25,27]. Among ambulatory HIV-infected patients with CKD, the prevalence of DM and HTN was 20% and 55%, respectively [20]. In analysis of more than 4000 patients enrolled in the EuroSIDA cohort, DM and HTN were reported as risk factors for the progression of CKD [23]. Several other studies have suggested that accelerated kidney disease progression in patients with HIV infection and comorbid DM [11,28,29]. Specifically, in a John Hopkins Cohort of more than 650 HIV patients, CKD patients with DM had a significant decrease in eGFR as compared to CKD patients without DM [28]. In two other cART-treated cohorts, comorbid DM was considered a strong risk factor for a decline in eGFR (−5.6ml/min/1.73m^2^ per year) as well as progression to ESRD in both blacks and whites. Finally, concurrent DM was also shown to increase the risk of tenofovir-associated nephrotoxicity [30].

Although these studies suggest that HIV infection may accelerate the progression of CKD related to comorbid DM and HTN, the mechanism behind this association has yet to be described. Specifically, translational studies are needed to demonstrate whether local HIV viral gene expression can potentiate these other forms of kidney disease. For example, it is not known whether a murine model of concurrent HIV gene expression and DM would progress to CKD at a faster rate than a murine model of either disease alone. Additionally, we need to determine the true spectrum and progression of disease by conducting longitudinal studies in patients with comorbid HIV and DM, compared to those with either disease alone. Next-generation sequencing could be performed from kidney biopsy tissue obtained from these patients to determine if there is a differential expression of genes or pathways that may explain the increased progression of kidney disease in this population. This research will help to determine whether HIV patients with CKD should be treated with cART regardless of the etiology.

## HIV and Race

Analysis from the USRDS revealed that approximately 90% of patients with ESRD attributed to HIVAN are of black race. Data from the AIDS link to the Intravenous Experience (ALIVE) study and the John Hopkins HIV Cohort Study revealed a 31-fold increase in ESRD in black patients as compared to white patients with HIV [28,33]. Not only is the incidence of CKD higher in black HIV patients, but the progression to ESRD seems to be increased as well. In a longitudinal study of more than 2 million U.S. Veterans, the incidence of ESRD among black HIV patients was shown to be 10 times higher than that of white HIV patients [34]. More than half of these ESRD cases were attributed to a non-HIVAN diagnosis. Furthermore, the aggressive nature of kidney disease among black patients is highlighted in an observational study from the John Hopkins HIV Clinical Cohort of more than 4000 HIV patients [33]. Although the incidence of CKD was only two fold higher among black HIV patients, the decline in eGFR was 6-fold more rapid. This lead to a dramatic 18-fold increase in the likelihood of progression to ESRD in black as compared to white patients with HIV [33]. Also, in a subgroup analysis of the available kidney biopsy data from this cohort, black HIV patients were reported to progress much faster than white HIV patients regardless of the etiology of kidney disease. Taken together, these studies suggest that the effect of race cannot be solely attributed to HIVAN. The increased propensity to HIV-related kidney disease in patients of African descent may be partially explained by recently discovered polymorphisms in apolipoprotein L-1 (APOL1) on chromosome 22 in Yoruban Africans [35,36]. These variants of APOL1 appear to be protective against African Sleeping Sickness compared to wild-type APOL1 [35]. This hypothesized survival advantage of APOL1 variants suggests a potential mechanism for increased incidence and prevalence of kidney disease in patients of African descent. More recently, Kopp et al. estimated that individuals with two APOL1 risk alleles had a 50% lifetime risk of developing HIVAN [37]. This evidence warrants future clinical trials to determine whether APOL1 could be used in clinical practice to identify high-risk individuals predisposed to kidney disease.

## HIV and HCV

Approximately 15–30% of HIV patients are co-infected with HCV, and an estimated 10 million people are co-infected with HIV and HCV worldwide [38]. In an early case series of 12 intravenous drug users (IVDU) co-infected with HIV and HCV, 11 had immune complex related glomerulonephritis (ICGN). The spectrum of ICGN ranged from membranoproliferative GN (MPGN) to membranous nephropathy [39]. Similarly, MPGN was the predominant diagnosis in HIV-HCV co-infected individuals in other biopsy series [40,43].

Epidemiologic data suggest that HCV co-infection is also associated with increased risk of CKD and CKD progression in HIV-infected individuals, although the contribution of HCV-related ICGN is not known. In a systematic review and meta-analysis of 12 clinical trials and observational studies, pooled analysis demonstrated that HCV co-infection was associated with an increase in proteinuria and CKD by 15% and 50%, respectively [42]. As with most meta-analyses, this study was limited by heterogeneity of individual study design and quality of available studies. In a subsequent analysis of data from WIHS, women with baseline CKD and HCV-HCV co-infection had a more a significant reduction in eGFR as compared to those with HIV alone [41]. Similarly, in a cohort of more than 9000 U.S. Veterans, a significant increase in CKD was observed in HCV co-infected individuals as compared to those with HIV alone (14% vs. 11%) [44]. Overall mortality was also significantly higher among HIV-HCV co-infected veterans [44], as observed in other studies [43]. Although not proven, the worse outcomes in HIV-HCV co-infected individuals has been hypothesized to reflect complications from liver disease or impaired immunological or virological response in the setting of co-infection. Future studies should consider the mechanisms by which HCV co-infection promotes CKD risk in HIV-infected individuals and determine whether successful treatment of HCV attenuates that risk. Such studies will require close collaboration between clinical and laboratory scientists in order to address potential mechanisms.

## HIVICK

An immune complex mediated disease (HIVICK) has also been described in certain HIV-infected populations independent of HCV co-infection. Early studies suggested that HIVICK predominantly occurs in white HIV patients [45,46]. In contrast, a South African study with all black HIV patients and no evidence of HCV, recently identified HIVICK in 21% of all biopsied patients. In this cohort, classic HIVAN accounted for less than 1/3 of all HIV-related kidney disease, suggesting that HIVICK may be an important cause of CKD in the cART era, regardless of race [47]. The contribution of HIV infection to the development of HIVICK is not known, and should be considered in future studies. Because there is no available animal model for the study of HIVICK, mechanistic studies will depend on collaboration with clinical investigators or clinicians who can provide access to kidney biopsy tissue from patients with this diagnosis.

## HIV and Combination Antiretroviral Therapy

Although widespread use of cART has clearly reduced the incidence of HIVAN since the mid 1990s [24,48,49], there is increasing evidence that nephrotoxicity associated with cART can also contribute to HIV-related kidney disease. Antiretroviral agents can lead to a wide variety of nephrotoxic effects, including crystal induced obstruction, tubular toxicity, acute interstitial nephritis, Type B Lactic Acidosis, and electrolyte changes [50]. Although the nephrotoxicity profiles of certain ARTs have been clearly demonstrated, the impact of long-term exposure on CKD and ESRD remains unclear. In 2005, the Strategies for Management of ART (SMART) study revealed that interruptions in the use of cART as compared to continuous use contributed to higher rates of renal adverse events [51]. However, with longer follow-up, additional events occurred in the group that received continuous rather than intermittent therapy, suggesting that continuous cART may have delayed but not prevented the progression of CKD [52]. In a smaller retrospective study of 89 biopsied patients, the use of cART in non-HIVAN kidney disease did not slow the progression to ESRD [17]. Similarly, in cohort of U.S. Veterans, the initial use of cART reduced the eGFR decline, but with time and durable viral suppression, patients continued to lose kidney function at the rate of −1.9ml/min/1.73m^2^ per year [29].

Although the main treatment for HIVAN has been cART, it is unclear if other types of HIV-related kidney disease benefit from the use of cART. The ongoing NIH-funded Strategic Timing of Antiretroviral Treatment (START) study, investigating the impact of early cART initiation on AIDS and non-AIDS conditions including CKD, is currently underway [www.clinicaltrials.gov].

## Protease Inhibitors

Indinavir was one of the initial protease inhibitors (PI) used in combination with other ART in the treatment of HIV. Although the use of Indinavir has decreased in the United States with the advent of other protease inhibitors, it is still commonly used in resource-limited settings. Indinavir, a drug with significant urinary excretion [53], is known to cause nephrolithiasis and interstitial nephritis [54–56], and with chronic use, can significantly reduce kidney function [21].

Nephrolithiasis and interstitial nephritis have also been reported in association with the newer PI atazanavir [57–60]. Approximately 7% of atazanavir is excreted in the urine unchanged after a single 400mg dose [61], and this drug has an increased tendency to precipitate due to its poor solubility at physiological urine pH [62]. In addition to case reports of atazanavir-associated nephrolithiasis [60], data from the EuroSIDA cohort demonstrated that patients on atazanavir had a 21% increased risk of developing CKD, which improved with the cessation of the drug [23]. Further studies are needed to determine the impact of chronic atazanavir use on the progression of HIV-related kidney disease.

Finally, the use of ritonavir as a boosting agent in combination with indinavir or tenofovir has been shown to increase nephrotoxicity [63,64], most likely as a result of drug interactions rather than a direct toxic effect of ritonavir.

## Nucleoside Reverse Transcriptase Inhibitors

Nucleoside analogues such as lamivudine and didanosine are excreted in the urine and therefore must be dose adjusted in the setting of CKD [8]. Furthermore, chronic use can lead to mitochondrial dysfunction leading to Type B Lactic Acidosis [65,66]. The evidence for proximal tubule dysfunction or worsening renal function has been minimal in comparison to nucleotide reverse transcriptase inhibitors [67].

## Nucleotide Reverse Transcriptase Inhibitors

Tenofovir Disoproxil Fumarate is a nucleotide reverse transcriptase inhibitor that has been widely used in the United States since the initial premarketing trials in 2004 [68–70]. Tenofovir is excreted in the urine by glomerular filtration as well as via tubular secretion. Studies have demonstrated these nucleotide analogues including tenofovir enter tubular epithelial cells via organic anionic transporters OAT1 and OAT3 and interact with multidrug-resistance-associated protein MRP4 on the luminal membrane for tubular secretion [71]. Although not well characterized, it has been suggested that the intracellular accumulation of nucleotide analogues may lead to mitochondrial depletion or apoptosis of proximal tubular epithelial cells, thereby resulting in tubular dysfunction [72,73].

The initial pre-marketing trials in 2004 did not reveal a significant decrease in kidney function with tenofovir use [68]. The absolute rates of drug discontinuation caused by renal dysfunction have ranged from 0–2% [67,78–81]. An observational study of more than 10,000 HIV patients found that tenofovir use was associated with an increase in serum Cr ≥ 0.5 and Cr ≥ 2 in 2.2% and 0.6%, respectively [82]. Additionally, only 0.5% of the serious adverse events were attributed to tenofovir use [82].

However, shortly after its market release and the recognition of acute nephrotoxicity in a minority of patients, observational studies reported an association with declining eGFR [23,28,75,76]. In a study of 658 patients in the John Hopkins Cohort, a 4% relative decline in creatinine clearance was reported with tenofovir use [28]. In the last five years, several additional observational studies have concluded that tenofovir use is associated with an accelerated decline in eGFR [76,83,84]. Despite the heterogeneity associated with design of the studies, a systematic review and meta-analysis of 17 studies found that tenofovir was associated with a small but a significant loss in eGFR (3.9ml/min/1.73m^2^) [85]. More recently, a retrospective study of 1647 HIV patients revealed that tenofovir was associated with a significant decline in eGFR 104 weeks after initiation of treatment. The greatest effect was observed in patients with eGFR > 80ml/min/1.73m^2^ [30]. Finally, in the EuroSIDA study, a multicenter prospective study of more than 16,000 patients, tenofovir use was associated with a 16% increase in CKD. This risk increased to 41% when tenofovir was used in combination with atazanavir [23]. It has been hypothesized that the lack of tenofovir-associated renal dysfunction in earlier clinical trials may have been due to study design, exclusion of patients with underlying CKD, or exclusion of patients on other nephrotoxic medications [73]. New expert guidelines from the Infectious Disease Society Guidelines are anticipated to update clinicians on frequency of monitoring kidney function as well as the use of tenofovir in CKD.

Future studies should focus on the identification of individuals who are at increased risk for cART nephrotoxicity. Better understanding of the mechanisms underlying the potential toxicity of tenofovir and atazanavir may help to identify risk factors and approaches to prevent or minimize toxicity.

## Conclusion

In this review, we describe the changing epidemiology of HIV-related kidney disease. In 2012, health care providers will manage an increasing HIV population with CKD and ESRD. Although the course of the disease may be less aggressive than HIVAN, the wide spectrum of kidney disease makes diagnosis as well as management extremely challenging. Furthermore, questions still remain about whether patients with CKD secondary to non-HIVAN related kidney disease should be treated with cART. It is evident that with increased survival in the HIV population, comorbidities such as DM, HTN, HCV coinfection, and chronic cART use will play a crucial role in the epidemiology of kidney disease. Although further well-designed randomized clinical trials are required to clearly understand the associations, translational research is also important to characterize the mechanisms through which these comorbidities contribute to HIV-related kidney disease.

## Figures and Tables

**Table 1:** Spectrum of Kidney Disease in HIV-infected Individuals.

HIV-associated nephropathy (HIVAN)
Immune complex-mediated kidney diseases
HIV immune complex kidney disease (HIVICK)
Membranoproliferative glomerulonephritis, with or without HCV co-infection
Membranous nephropathy, with or without HBV co-infection
IgA Nephropathy
Non-collapsing focal segmental glomerulosclerosis
Minimal change disease
Arterionephrosclerosis
Diabetic Nephropathy

**Table 2:** Chronic kidney disease (CKD) in HIV-infected individuals (United States and Europe).

Reference	Study Design	N	GFR estimate^a^	Confirmed GFR <60	Proteinuria assessed	Prevalence of CKD	Incidence of CKD
Choi et al. 2007	Cohort Study	15,135	MDRD	No	No	7.1%	1.4% (ESRD^d^)
Wyatt et al. 2007	Cross-sectional	1,239	MDRD	No	Yes	15.5%	-
Lucas et al. 2008	Cohort Study	4,259	MDRD	Yes	Yes	6.7%	-
Campbell et al. 2009	Cross-sectional	3,439	MDRD	Yes	No	2.4%	-
Choi et al. 2009	Cohort Study	615	MDRD	No	No	7.8%	-
Mocroft et al. 2010	Cohort Study	6,843	CG^c^	Yes	No	4.1%	3.3%
Flandre et al. 2011	Cohort Study	7,378	MDRD	Yes	No	-	4.7%

a GFR: glomerular filtration rate

b MDRD: Modification of Diet in Renal Disease

c CG: Cockcroft-Gault creatinine clearance

d ESRD: end-stage renal disease

## Abbreviations

cART: Combination Antiretroviral Therapy
HIVAN: HIV-Associated Nephropathy
HIVICK: HIV Immune Complex Kidney Disease
CKD: Chronic Kidney Disease
ESRD: End-Stage Renal Disease
